# Prognosis prediction using significant pathological response following neoadjuvant immunotherapy in resectable non-small-cell lung tumors: a meta-analysis

**DOI:** 10.3389/fsurg.2024.1500593

**Published:** 2024-11-22

**Authors:** Fang Nie, Ying Wang, Wanting Shi, Liru Zhu, Jing Hao, Rancen Tao

**Affiliations:** ^1^ Thoracic Oncology Department, Baotou Cancer Hospital, Baotou, Inner Mongolia, China; ^2^ Oncology and Palliative Care Department, Baotou Cancer Hospital, Baotou, Inner Mongolia, China; ^3^ Thoracic Oncology Surgery Department, Baotou Cancer Hospital, Baotou, Inner Mongolia, China

**Keywords:** resectable non-small-cell lung tumors, prognosis prediction, pathological response, programmed death-ligand 1, neoadjuvant immunotherapy, stage

## Abstract

**Background:**

A meta-analysis study was done to figure out how to predict the prognosis of people with resectable non-small-cell lung cancer (NSCLC) who had a significant pathological response following neoadjuvant immunotherapy.

**Methods:**

Up until August 2024, a comprehensive literature study was completed, and 2,386 connected studies were revised. The 35 selected studies included 3,118 resectable non-small-cell lung tumor participants at the beginning of the study. Using dichotomous techniques and a fixed or random model, the odds ratio (OR) and 95% confidence intervals (CIs) were used to assess the prediction using significant pathological response following neoadjuvant immunotherapy in resectable NSCLC.

**Results:**

Individuals with resectable NSCLC had significantly higher major pathological response when comparing neoadjuvant chemo-immunotherapy to neoadjuvant chemotherapy (OR, 5.07; 95% CI, 4.09–6.27, *p* < 0.001), objective response rate to non-objective response rate (OR, 7.02; 95% CI, 4.28–11.50, *p* < 0.001), and programmed death-ligand 1 ≥1% to programmed death-ligand ≤1% (OR, 2.49; 95% CI, 1.44–4.30, *p* = 0.001). However, no significant difference was found in major pathological response between stage III and stage I-II (OR, 1.43; 95% CI, 0.88–2.33, *p* = 0.15), and squamous cell cancer and non-squamous cell cancer (OR, 1.35; 95% CI, 0.95–1.92, *p* = 0.09) in individuals with resectable NSCLCs.

**Conclusion:**

Individuals with resectable NSCLCs had significantly higher major pathological response when comparing neoadjuvant chemo-immunotherapy to neoadjuvant chemotherapy, objective response rate to non-objective response rate, and programmed death-ligand 1≥1% to programmed death-ligand 1 ≤1%, however, no significant difference was found between stage III and stage I-II, and squamous cell cancer and non-squamous cell cancer. To validate this discovery, more research is required since most of the selected studies had a low sample size, and caution must be implemented when interacting with its values.

## Introduction

One of the most prevalent and fatal malignancies worldwide is lung cancer (1). The cornerstone of care for non-small cell lung cancer (NSCLC) that is both locally progressed and in its early stages is still surgical resection. Nevertheless, despite curative resection, 30%–55% of patients with NSCLC have recurrence and ultimately pass away from their illness, even in the early stages of the disease (2, 3). According to a meta-analysis of NSCLC, adding chemotherapy to neoadjuvant care could result in a 5% increase in survival after five years (4). Immune checkpoint inhibitors that target the programmed cell death protein 1/programmed death-ligand 1 (PDL 1) axis are currently the mainstay of treatment for metastatic NSCLC, either used alone or in conjunction with chemotherapy. Numerous phase 2 neoadjuvant immunotherapy trials have demonstrated positive results, indicating that immune checkpoint inhibitors, either in combination with chemotherapy or on their own, can significantly minimize the growth of cancers that have spread locally or enhance their pathological regression (5). In the neoadjuvant setting, the major pathological response (MPR), which is defined as 10% or less viable tumor, ranges from 19% to 45% with a single drug and varies from 33% to 83% when paired with chemotherapy (6). Adjuvant nivolumab with chemotherapy demonstrated statistically significant improvements in pathological complete response rate, MPR rate, and event-free survival when compared to chemotherapy alone (6). The transition of possibly successful treatment to clinical practice may be delayed by the requirement for an extended follow-up time, even though the gold standard of outcome measurement for phase 3 trials which is overall survival. It has also been suggested to use a MPR as a potential surrogate endpoint to quickly assess the clinical effectiveness of neoadjuvant chemotherapy. From 2008 to 2012, 151 patients with NSCLC were managed with neoadjuvant chemotherapy and then experienced total surgical resection (7). Multivariable analysis of the data showed that MPR was related to long-term overall survival. According to Hellman et al. (8), MPR was highly correlated with increased survival, accurately depicted the effect of treatment, and adequately measured the extent of treatment benefit on survival. As of yet, the immunotherapy age has not shown the evidence-based validity of MPRs.

### Objectives

This time, we used a meta-analysis to assess the reliability of MPRs as a proxy for survival following neoadjuvant immunotherapy.

## Methods

### Eligibility criteria

To provide an overview, the studies that showed the prognosis prediction using significant pathological response following neoadjuvant immunotherapy in resectable NSCLCs were picked (9).

### Information sources

**Figure 1:**
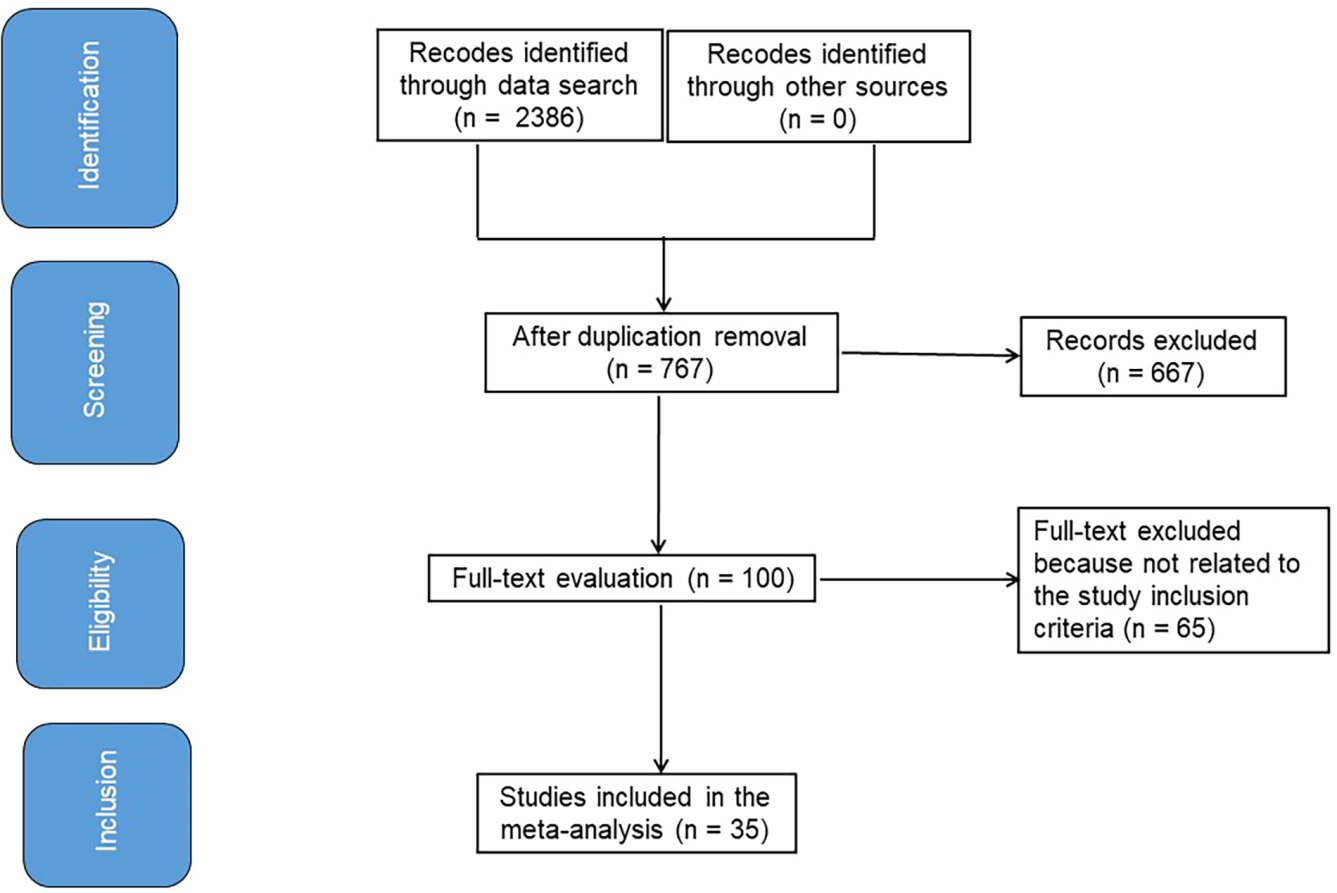
Shows a procedure flowchart for the research.

Figure 1 symbolizes the entirety of the study. When the following inclusion criteria were satisfied, the literature was incorporated into the study (10, 11):

1. The study was a randomized controlled trial (RCT), observational, prospective, or retrospective study.
2. The people who were chosen for investigation have resectable NSCLCs.
3. MPR was integrated into the intervention.
4. The study made a distinction of the prognosis prediction using significant pathological response following neoadjuvant immunotherapy in resectable NSCLCs.

Studies that did not check the possessions of the prognosis prediction using significant pathological response following neoadjuvant immunotherapy in resectable NSCLCs, studies on MPR in individuals without neoadjuvant immunotherapy, and studies with no comparison significance were also omitted (12, 13).

### Search strategy

A search protocol process was identified using the PICOS view, and we defined it as follows: the “population” consisted of people with resectable NSCLCs, P; MPR was the “intervention” or “exposure,” and the “comparison” involved correlation between MPR and different patients’ variables; the “outcome” was the effect on MPR; and the “research design” was without boundaries (14).

We have thoroughly searched the databases of Google Scholar, Embase, the Cochrane Library, PubMed, and OVID through August 2024 using a set of keywords and additional terms as shown in Table 1 (15, 16). To prevent the inclusion of a study that was unable to establish a link between the effects of MPR in resectable NSCLCs and its prognosis prediction, the replications of the papers were eliminated, assembled into an EndNote file, and their titles and abstracts were once again assessed (17, 18).

**Table 1:** Database search strategy for inclusion of examinations .

Database	Search strategy
Google Scholar	#1 “resectable non-small-cell lung tumors” OR “prognosis prediction”
	#2 “pathological response” OR “programmed death-ligand 1” OR “neoadjuvant immunotherapy” OR “stage”
	#3 #1 AND #2
Embase	#1 “resectable non-small-cell lung tumors” /exp OR “prognosis prediction” /exp OR “neoadjuvant immunotherapy”
	#2 “pathological response”/exp OR “programmed death-ligand 1”/exp OR “stage”
	#3 #1 AND #2
Cochrane library	#1 (resectable non-small-cell lung tumors):ti,ab,kw (prognosis prediction):ti,ab,kw (neoadjuvant immunotherapy):ti,ab,kw (Word variations have been searched)
	#2 (pathological response):ti,ab,kw OR (programmed death-ligand 1):ti,ab,kw OR(stage):ti,ab,kw (Word variations have been searched)
	#3 #1 AND #2
Pubmed	#1 “resectable non-small-cell lung tumors"[MeSH] OR “prognosis prediction"[MeSH] OR “neoadjuvant immunotherapy” [All Fields]
	#2 “pathological response”[MeSH Terms] OR “programmed death-ligand 1"[MeSH] OR “stage ”[All Fields]
	#3 #1 AND #2
OVID	#1 “resectable non-small-cell lung tumors”[All Fields] OR “prognosis prediction” [All Fields] OR “neoadjuvant immunotherapy” [All Fields]
	#2 “pathological response”[ All fields] OR “programmed death-ligand 1”[All Fields] or “stage”[All Fields]
	#3 #1 AND #2

### Selection process

The meta-analysis method was then used to organize and assess the method that followed the epidemiological proclamation (19, 20).

### Data collection process

Some of the criteria utilized to gather data were the name of the first author, research data, research year, nation or region, population type, categories, quantitative and qualitative estimation methods, data sources, outcome estimation, medical and therapy physiognomies, and statistical analysis (21).

### Data items

When a study yielded differing values, we independently gathered the data founded on a valuation of prognosis prediction using significant pathological response following neoadjuvant immunotherapy in resectable NSCLCs.

### Research risk of bias assessment

Two authors looked into the opportunity for bias in the studies and the standard of approaches utilized in papers elected for supplementary analysis. The two authors (Fang Nie, and Ying Wang) conducted unbiased reviews of techniques used for each test.

### Effect measures

Sensitivity analysis was limited to studies that assessed and documented the prognosis prediction using significant pathological response following neoadjuvant immunotherapy in resectable NSCLCs. A subclass analysis was used to compare the correlation between MPRs and different patients’ variables in resectable NSCLCs individuals’ sensitivity.

### Synthesis methods

Using a dichotomous approach and a random or fixed-effect model, the odds ratio (OR) and a 95% confidence interval (CI) were determined. A range of 0%–100% was used to determine the I^2^ index. At 0%, 25%, 50%, and 75% of the data, respectively, there was no, low, moderate, and significant heterogeneity visible (22). To ensure that the exact model was used, additional structures that show a high degree of similarity with the related inquiry were also examined. The fixed-effect rose was an option if I^2^ was less than 50%; otherwise, the random effect was used (22). A subclass analysis was performed by splitting the original estimation into the previously specified consequence groups. A *p*-value of less than 0.05 was utilized in the analysis to define the statistical significance of differences across subcategories.

### Reporting bias assessment

Both quantitative and qualitative methods were employed to measure the bias in the investigations: the Egger regression test and funnel plots, which display the logarithm of the ORs against their standard errors. The presence of investigation bias was determined by *p* ≥ 0.05 (23).

### Certainty assessment

We looked at each *p*-value with two-tailed testing. Graphs and statistical analyses were created using Reviewer Manager Version 5.3 (The Nordic Cochrane Centre, the Cochrane Collaboration, Copenhagen, Denmark).

## Results

Out of 2,386 connected studies, 35 papers that were published between 2018 and 2024 and satisfied the inclusion criteria were selected for the study (24–58). Table 2 provides access to the findings of these inquiries. At the beginning of the studies that were used, there were 3,118 resectable NSCLC participants. There were between 8 and 740 subjects as a sample size.

**Table 2:** Qualities of the chosen studies for the meta-analysis.

Study	Country	Total	Neoadjuvant chemo-immunotherapy	Neoadjuvant chemotherapy	Clinical stage
Forde (24)	USA	21	8	13	I-IIIA
Lei et al. (25)	China	13	7	6	IIIA or IIIB-N2
Provencio et al. (26)	Spain	46	35	11	IIIA
Shu et al. (27)	USA	26	19	7	IIA-IIIA
Gao et al. (28)	China	37	8	29	IA–IIIB
Tao et al. (29)	China	36	17	19	IA–IIIB
Tfayli et al. (30)	Lebanon	11	3	8	IB-IIIA
Altorki et al. (31)	USA	57	36	21	I–IIIA
Cascone et al. (32)	USA	44	21	23	I-IIIA
Liang et al. (33)	China	20	10	10	IIB-IIIB
Duan et al. (34)	Italy	47	14	33	IIA-IIIB
Shen et al. (35)	China	37	29	8	IIB–IIIB
Eichhorn et al. (36)	Germany	14	4	10	II/IIIA
Rothschild et al. (37)	Switzerland	58	27	31	IIIA (N2)
Chen et al. (38)	Multi-centered	35	17	18	IIIA/IIIB
Forde et al. (39)	USA	358	179	179	IB-IIIA
Provencio et al. (40)	Spain	86	57	29	IIIA
Hou et al. (41)	China	55	31	24	IIIA or IIIB
Liu et al. (42)	China	170	79	91	IB-IIIB
Zhao et al. (43)	China	140	42	98	IB–IIIB
Chaft et al. (44)	USA	133	10	123	IB–IIIB
Tong et al. (45)	USA	30	13	17	IB-IIIA
Zhang et al. (46)	China	29	18	11	IB-IIIA
Wu et al. (47)	China	37	26	11	II-III
Chen et al. (48)	China	12	6	6	IIIA-IIIB
Fan et al. (49)	China	8	7	1	III
Rosner et al. (50)	USA	20	6	14	I-IIIA
Provencio et al. (51)	Spain	86	57	29	III
Fang et al. (52)	China	211	172	39	II-IIIA
Yang et al. (53)	China	50	23	27	IIIA-IIIB
Fei et al. (54)	China	167	66	101	II-IIIB
Lei et al. (55)	China	88	43	45	IIIA or IIIB
Heymach et al. (56)	USA	740	366	374	II-IIIB
Zhang et al. 2023 (57)	China	128	64	64	IB–IIIB(T3-4N2)
Mitsudomi et al. (58)	Japan	68	33	35	IB-IIIA
	**Total**	**3,118**	**1,545**	**1,565**	

**Figure 2:**
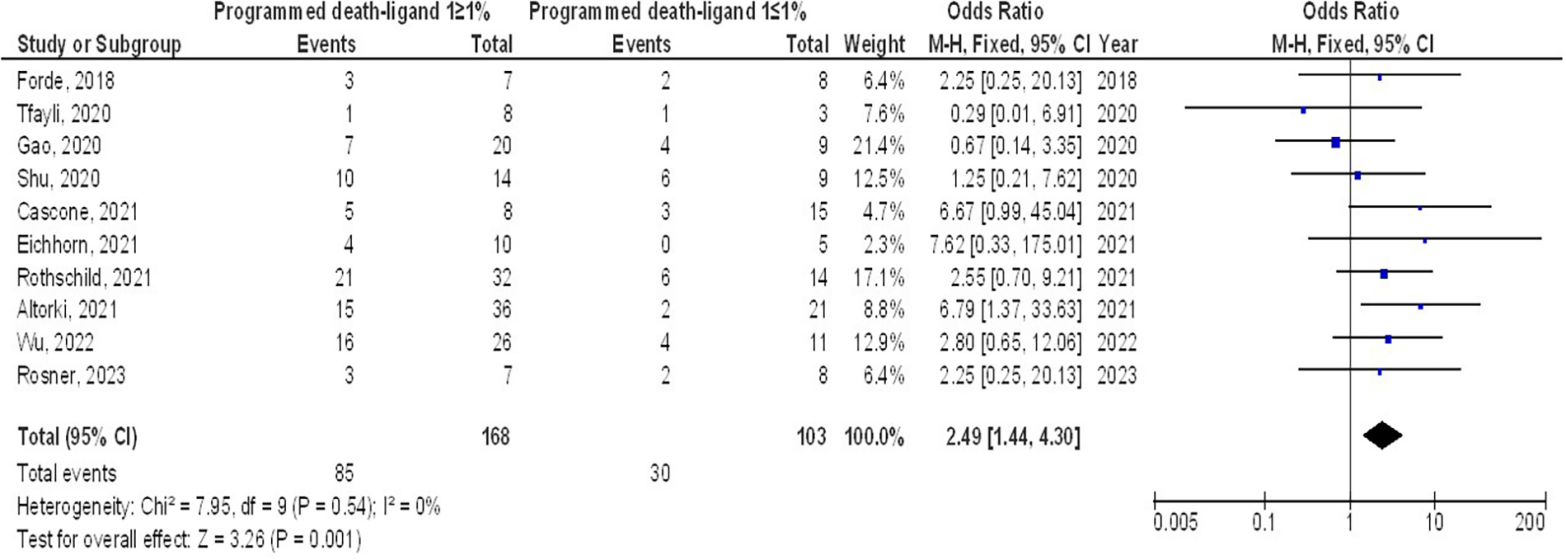
The neoadjuvant chemo-immunotherapy compared to neoadjuvant chemotherapy's forest plot influence on MPR in resectable NSCLC.

**Figure 3:**
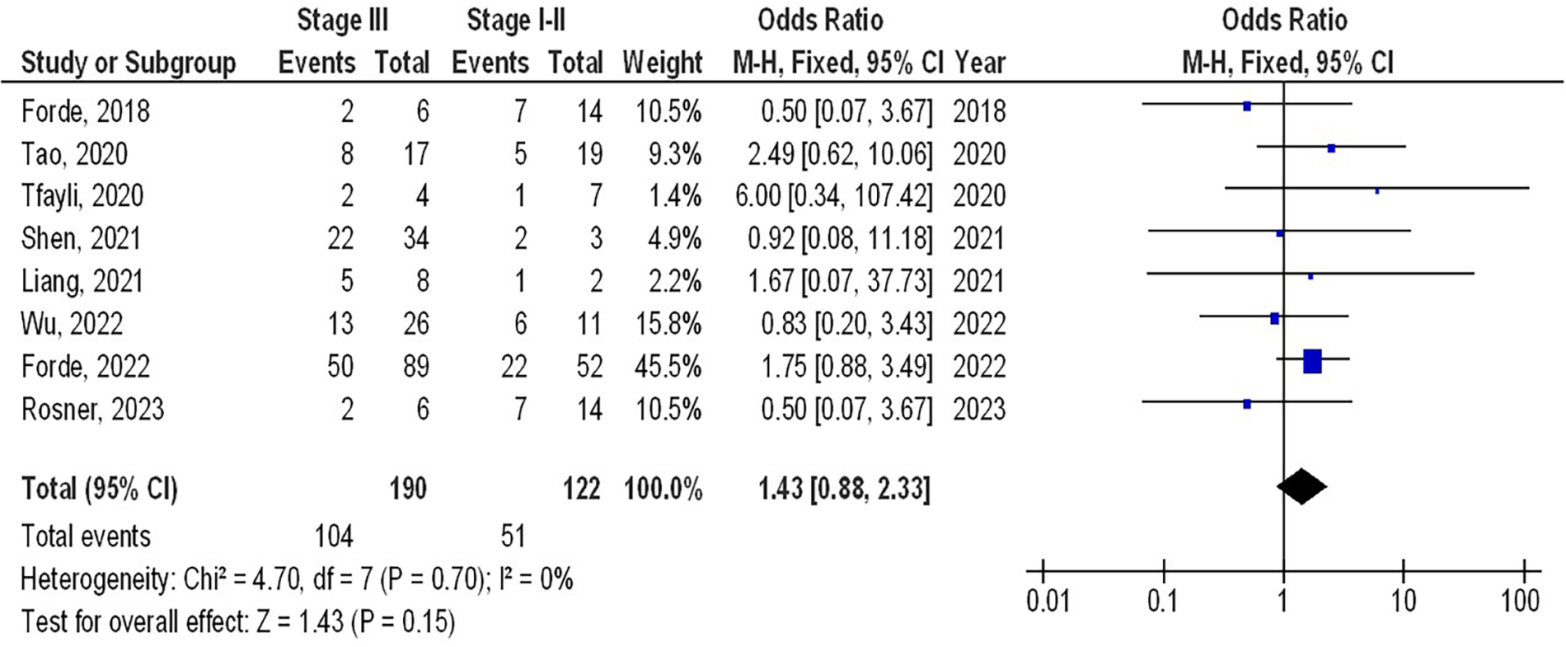
The objective response rate compared to non-objective response rate's forest plot influence on MPR in resectable NSCLC.

**Figure 4:**
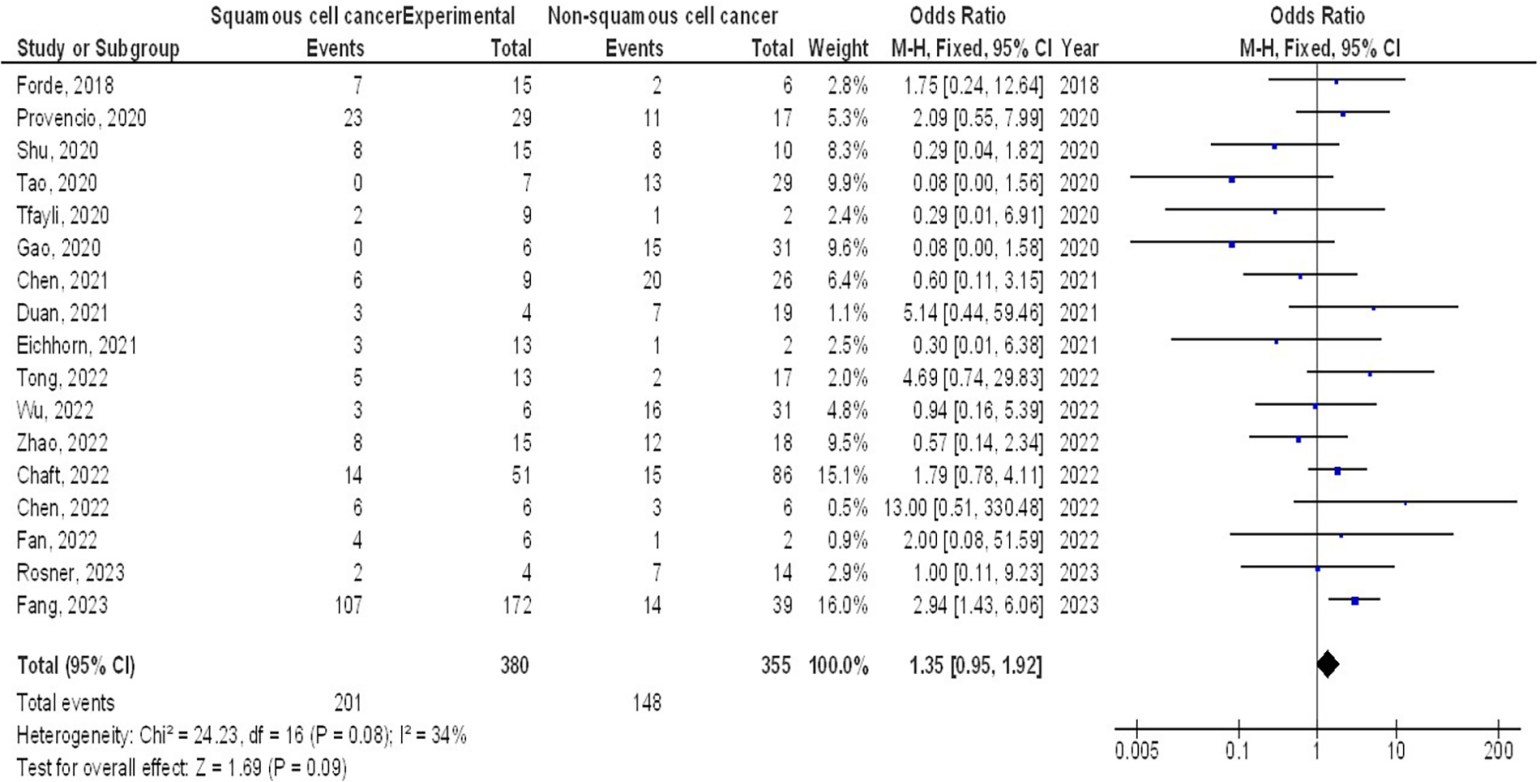
The PDL 1 ≥1% compared to PDL 1 ≤1%'s forest plot influence on MPR in resectable NSCLC.

As illustrated in Figures 2–4, Individuals with resectable NSCLCs had significantly higher MPR when comparing neoadjuvant chemo-immunotherapy to neoadjuvant chemotherapy (OR, 5.07; 95% CI, 4.09–6.27, *p* < 0.001) with low heterogeneity (I^2^ = 42%), objective response rate to non-objective response rate (OR, 7.02; 95% CI, 4.28–11.50, *p* < 0.001) with no heterogeneity (I^2^ = 19%), and PDL 1 ≥ 1% to PDL 1 ≤ 1% (OR, 2.49; 95% CI, 1.44–4.30, *p* = 0.001) with no heterogeneity (I^2^ = 0%).

**Figure 5:**
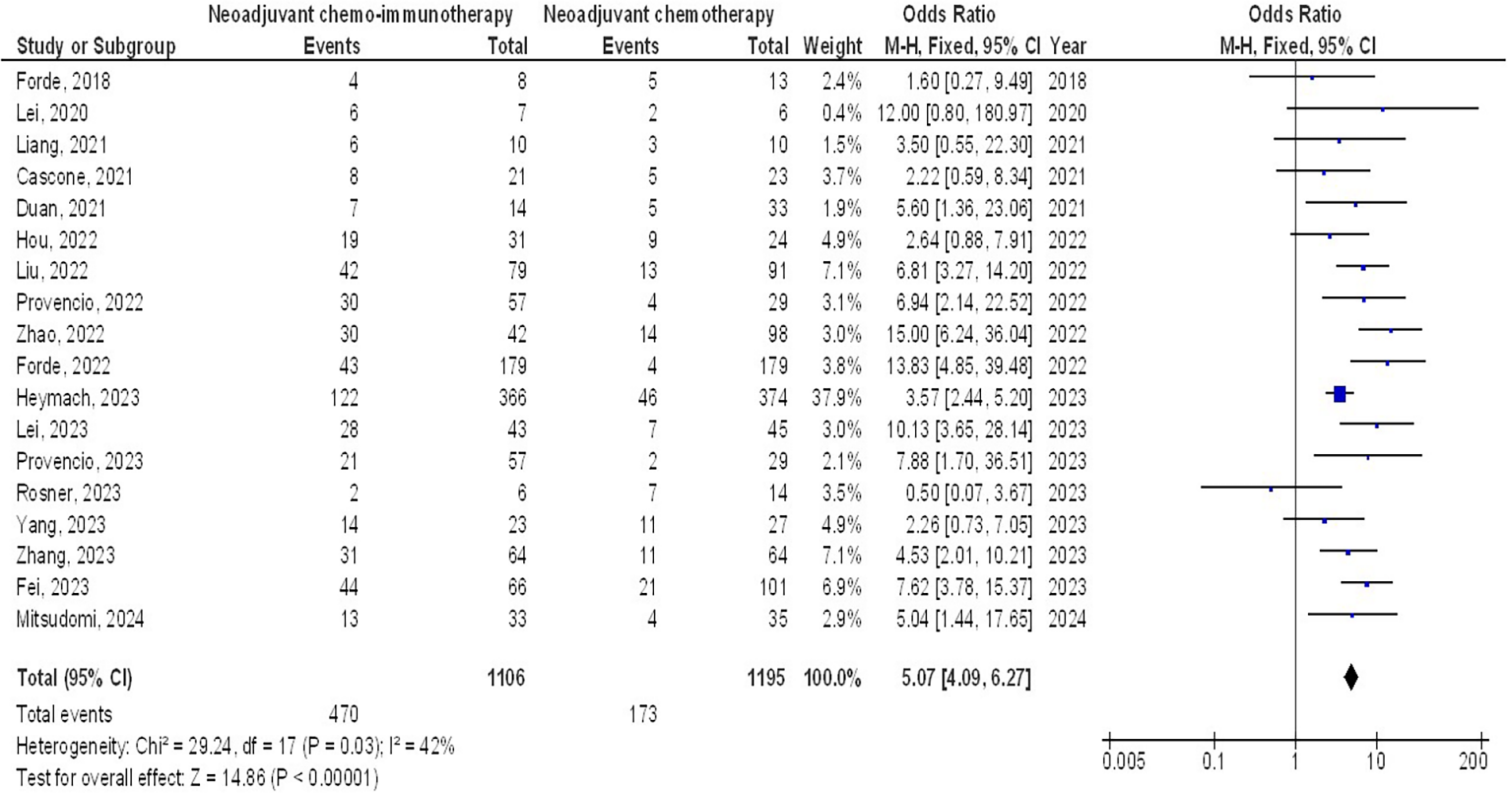
The stage III compared to stage I-iI's forest plot influence on MPR in resectable NSCLC.

**Figure 6:**
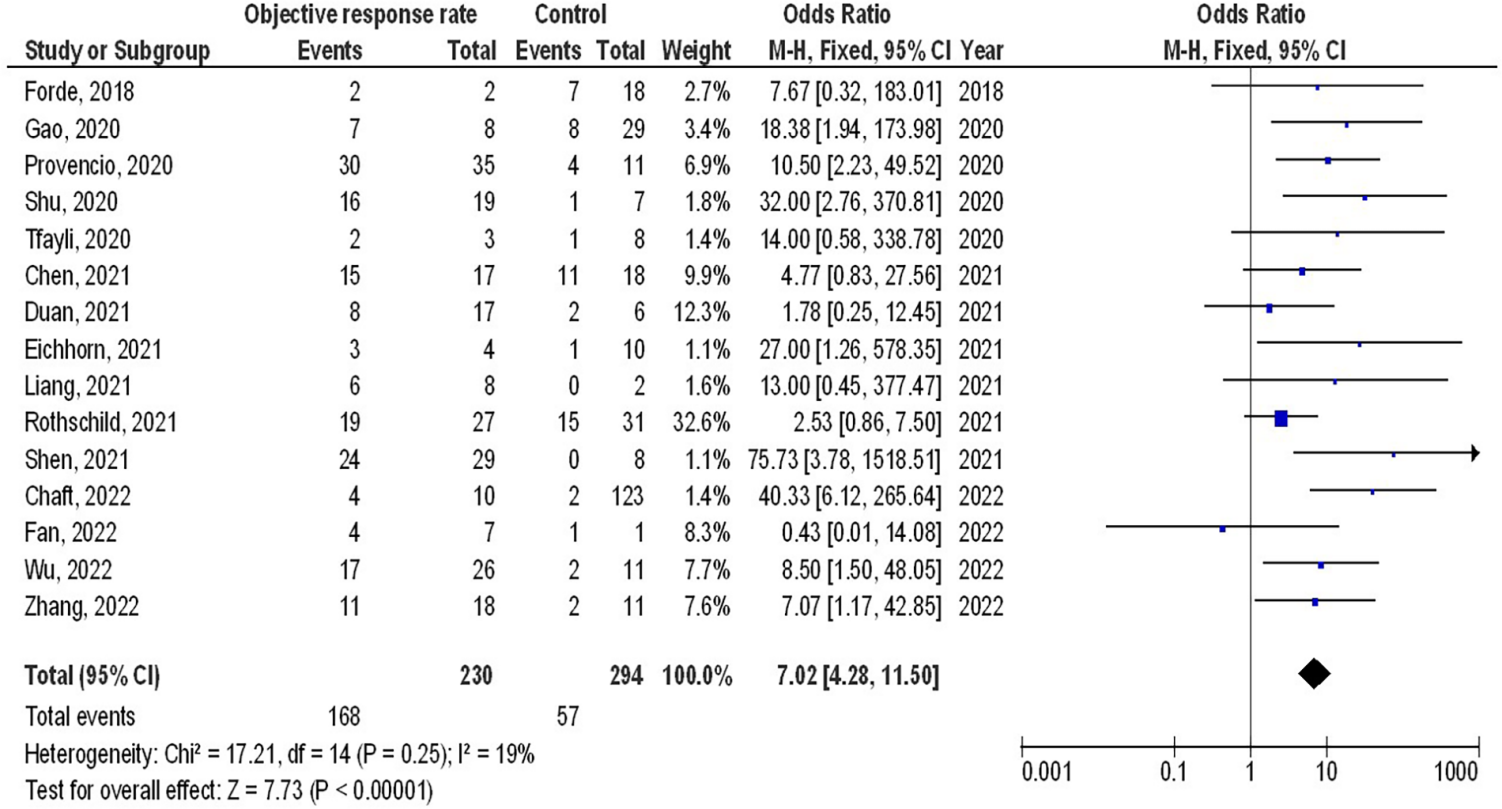
The squamous cell cancer compared to non-squamous cell cancer's forest plot influence on MPR in resectable NSCLC.

However, no significant difference was found in MPR between stage III and stage I-II (OR, 1.43; 95% CI, 0.88–2.33, *p* = 0.15) with no heterogeneity (I^2^ = 0%), and squamous cell cancer and non-squamous cell cancer (OR, 1.35; 95% CI, 0.95–1.92, *p* = 0.09) with low heterogeneity (I^2^ = 34%) in resectable NSCLCs, as shown in Figures 5, 6.

**Figure 7:**
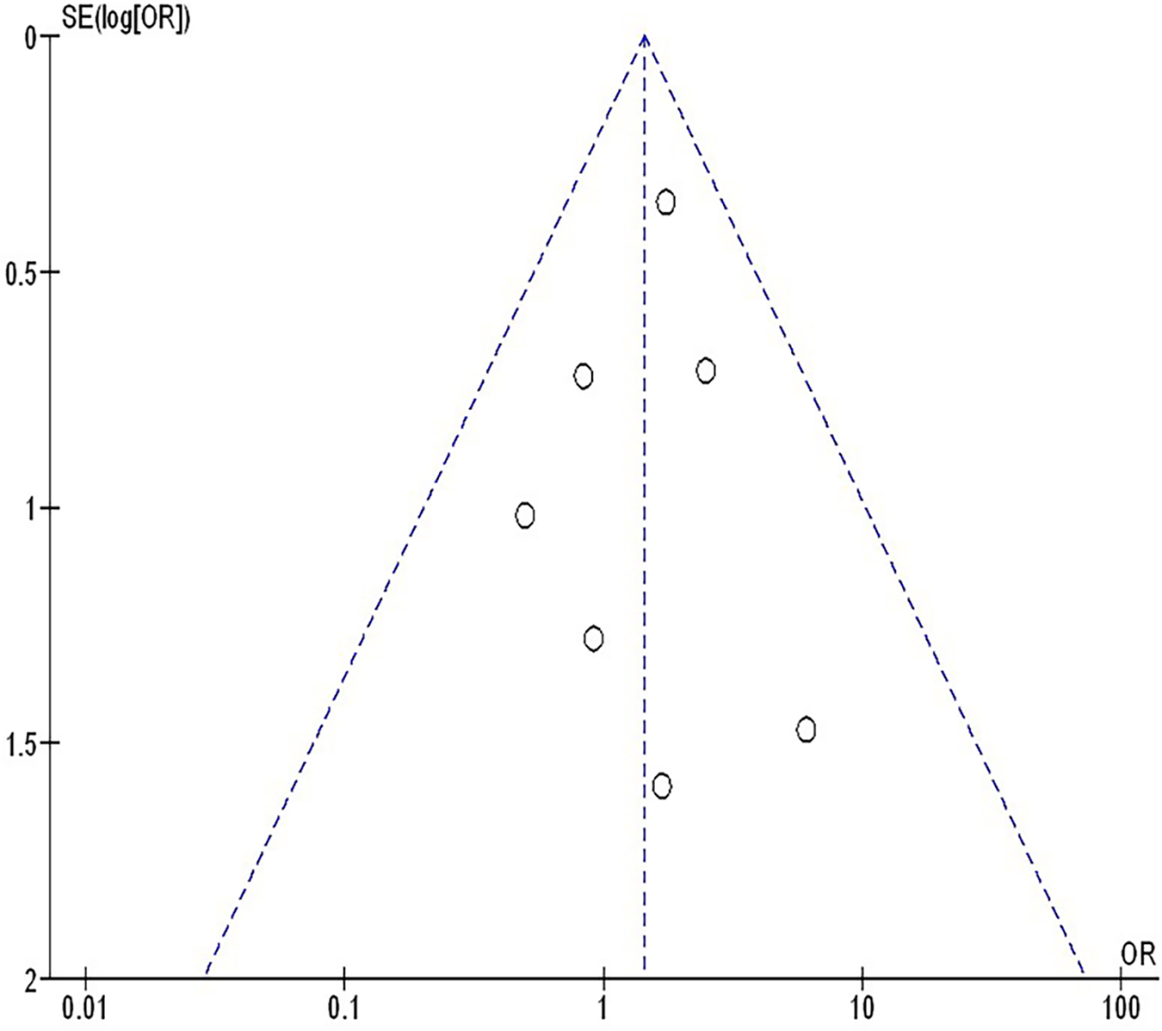
The neoadjuvant chemo-immunotherapy compared to neoadjuvant chemotherapy's funnel plot influence on MPR in resectable NSCLC.

**Figure 8:**
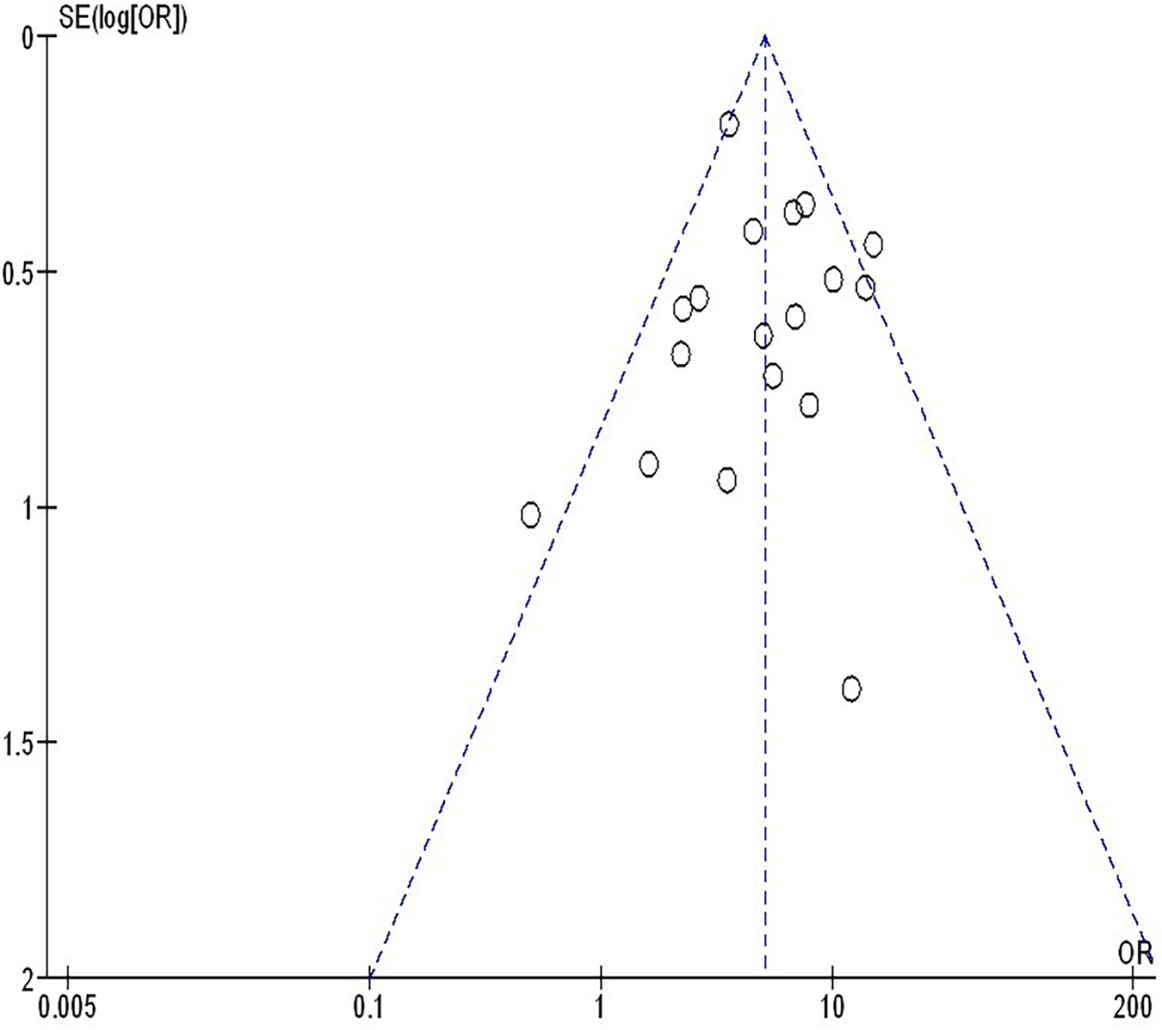
The objective response rate compared to non-objective response rate's funnel plot influence on MPR in resectable NSCLC.

**Figure 9:**
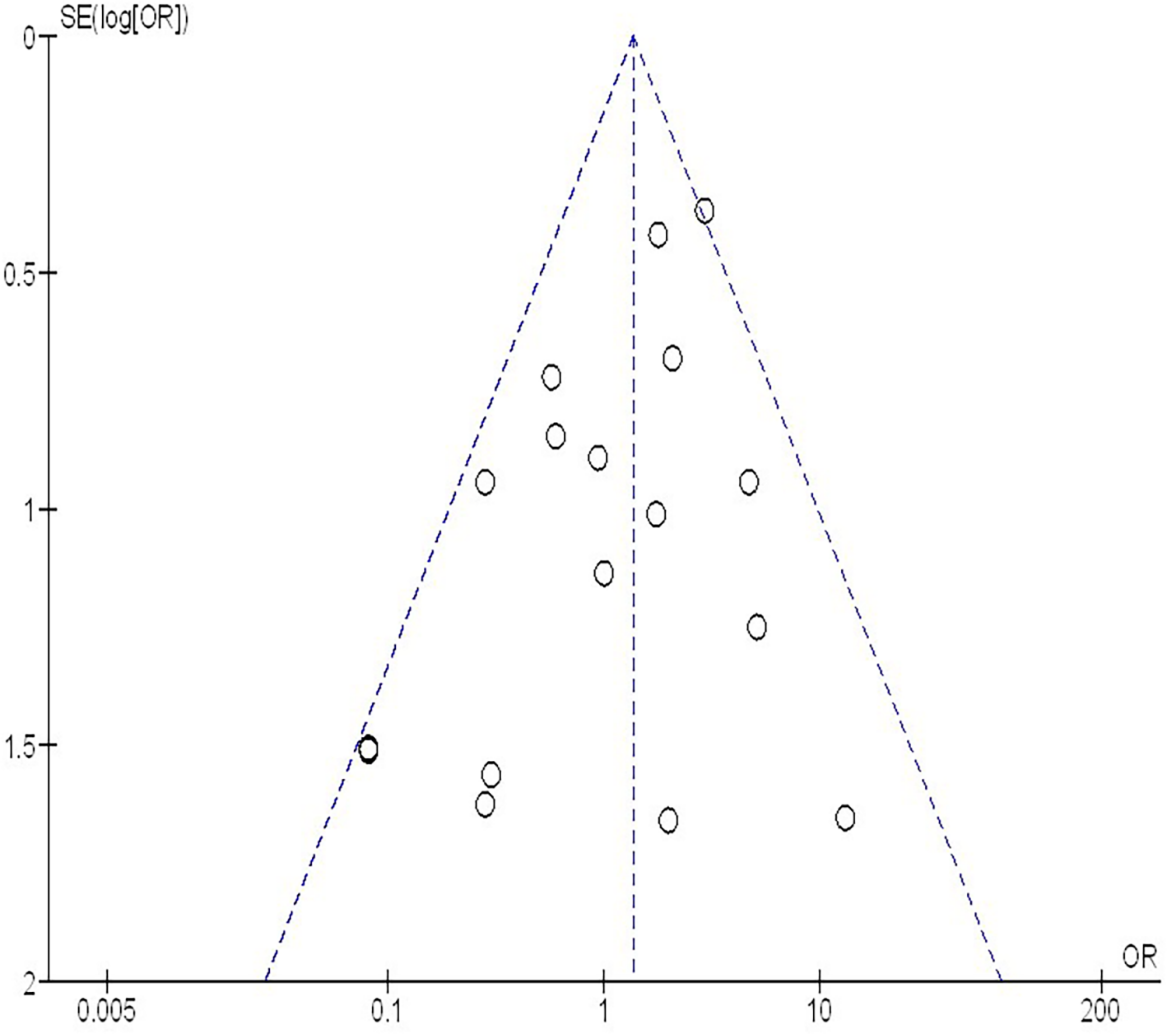
The PDL 1 ≥1% compared to PDL 1 ≤1%'s funnel plot influence on MPR in resectable NSCLC.

**Figure 10:**
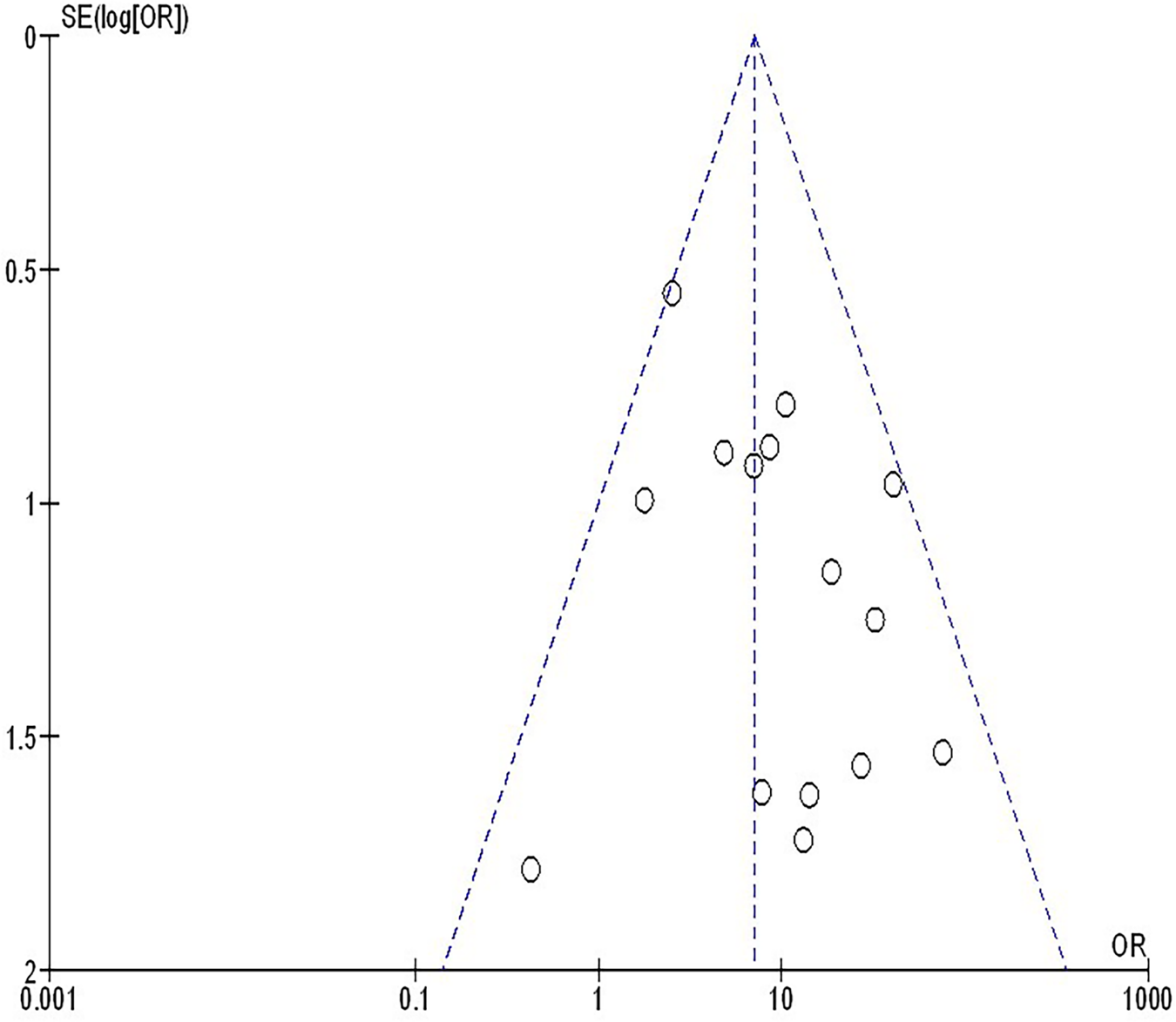
The stage III compared to stage I-iI's funnel plot influence on MPR in resectable NSCLC.

**Figure 11:**
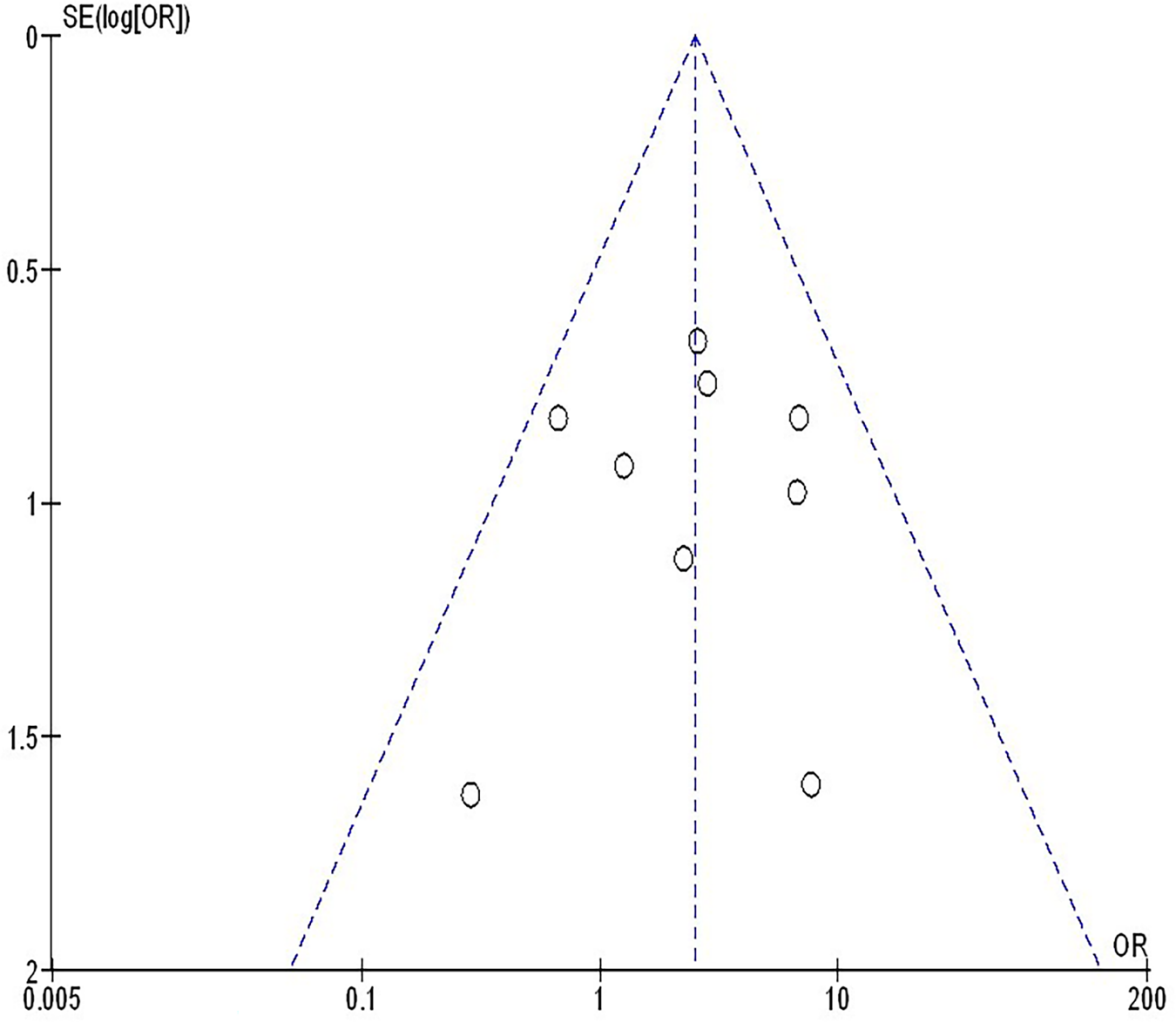
The squamous cell cancer compared to non-squamous cell cancer's funnel plot influence on MPR in resectable NSCLC.

The insufficiency of data, e.g., age, ethnicity, and gender, on comparative results precluded the application of stratified models to investigate the impacts of particular components. Using the quantitative Egger regression test and the visual interpretation of the funnel plot, no evidence of research bias was detected (*p* = 0.89) as shown in Figures 7–11. However, it was shown that there was no bias in the selective reporting and that the majority of concerned RCTs had poor technical quality.

## Discussions

3,118 resectable NSCLC participants were at the starting point of the studies that were utilized for the meta-analysis (24–58). Individuals with resectable NSCLCs had a significantly higher MPR when comparing neoadjuvant chemo-immunotherapy to neoadjuvant chemotherapy, the objective response rate to non-objective response rate, and PDL 1 ≥ 1% to PDL 1 ≤ 1%. However, no significant difference was found in MPRs between stage III and stage I-II, and squamous cell cancer and non-squamous cell cancer in resectable NSCLCs. To validate this discovery, more studies are required since most of the selected studies had a low sample size (27 out of 35 studies were <100 subjects), and thoughtfulness must be exercised when interrelating with its values. That would have an impact on how significant the evaluated assessments were (59–69).

Patients with resectable NSCLC may fare better with neoadjuvant treatment. Finding the ideal neoadjuvant strategy to attain a high response rate and manageable toxicity is still a challenge. The use of immune checkpoint inhibitors in early-stage NSCLC has gained attention as immunotherapy has lately emerged as a possible therapeutic approach for the disease. Due to the cancer's total antigen load before surgical resection, the administration of early immune checkpoint inhibitor therapy may elicit a profound pathological response (39, 70). 15% was the MPR rate after neoadjuvant chemotherapy, according to Brandt et al. (71) Neoadjuvant nivolumab produced a significant pathological response in 45% of individuals, according to the CheckMate 159 study (24). The efficacy of nivolumab in conjunction with carboplatin/paclitaxel as neoadjuvant therapy for patients with stage IIIa resectable NSCLC was assessed in the phase II RESECTABLE NON-SMALL-CELL LUNG CANCER research. Patients with locally advanced NSCLC may now have neoadjuvant chemo-immunotherapy as a novel option, according to a high MPR rate of 82.9% (26). In the phase III CheckMate-816 trial, it was found that nivolumab plus chemotherapy improved the MPR as a neoadjuvant treatment for resectable NSCLC by 36.9 vs. 8.9%, respectively, when compared to neoadjuvant platinum-based chemotherapy alone (39). The high rates of substantial pathological response may be explained by the synergistic action of immune checkpoint inhibitors and chemotherapy, with cytotoxic chemotherapy boosting the recognition of these drugs as immunotherapies (72, 73). Lack of surrogate endpoints of clinical success typically prevented innovative perioperative treatment options for resectable NSCLC from being widely accepted. A thorough assessment of the ongoing neoadjuvant therapy trials including patients with NSCLC is necessary, as pathological response has demonstrated a patient-level connection with survival in a variety of malignancies (74, 75). A combined analysis of two neoadjuvant chemotherapy studies revealed that pathological complete response was a favorable prognostic factor of overall survival (76). Five-year overall survival was 80.0% in the pathological complete response group compared to 55.8% in the non-pathological complete response group. Its use as a surrogate endpoint was severely limited, possibly due to the low pathological complete response rate following neoadjuvant therapies and the lack of appropriate data available for study. MPR appears to be more common than pathological complete response. MPR has been recognized as an additional predictor of survival in patients with NSCLC who underwent neoadjuvant chemotherapy, despite the lack of mediastinal downstaging assessment. Following neoadjuvant chemotherapy, Waser et al. observed that the main pathological response rate was 30% and that the histopathologic response was a strong predictor of overall survival. The MPR was suggested by the College of American Pathologists as one of the research endpoints for clinical trials including neoadjuvant immunotherapy for lung cancer (8). Nevertheless, there is still much to learn about the association between substantial pathological response and overall survival in patients with resectable NSCLC undergoing neoadjuvant immunotherapy. The MPR appeared to be a different measure of overall survival for individuals who underwent neoadjuvant chemotherapy for NSCLC. Neoadjuvant chemotherapy produced a good radiological response rate in the multicenter randomized trial MRC LU22/NVALT 2/EORTC 08012. On the other hand, no proof of any advantage in terms of overall survival was found. It was common to see differences between the pathological and radiographic assessments. When compared to traditional chemotherapeutic drugs, the tumor response patterns of immune agents may vary (77). With neoadjuvant chemo-immunotherapy, the incidence of radiographic partial response and complete response varied from 38 to 72% (26, 35, 37). Pseudo progression was initially reported in melanoma patients receiving ipilimumab treatment. It was defined as the radiologic advancement of the tumor burden followed by an objective response (78). According to certain research, immune checkpoint inhibitor-treated cancer types may have pseudoprogression. Conventional cytotoxic treatment does not usually produce this unusual effect. The traditional response evaluation criteria in solid tumors is still a valid and useful way to evaluate immunotherapy response in the clinic, even if additional radiologic criteria tailored specifically to immunotherapy have been developed (79). There was currently no agreement despite recent trials evaluating putative prognostic biomarkers for significant pathological response. Lung adenocarcinoma and lung squamous carcinoma are the two predominant subtypes that account for about 80% of instances of NSCLC (80). In comparison to adenocarcinoma, squamous cell cancer patients have comparatively greater MPR rates, according to several studies (26, 57). Based on several sizable prospective trials and the Lung Adjuvant Cisplatin Evaluation meta-analysis, patients with early stages of the illness (stages IB to II) are typically advised to undergo upfront resection and adjuvant chemotherapy (81). Which stages of NSCLC respond best to neoadjuvant immune checkpoint inhibitor therapy is yet unknown. It is crucial to evaluate pathological responses according to stages since this could enable better trial designs in the future for particular disease stages (73). According to the results of the CheckMate-816 trial (NCT02998528), patients with stage IIIA disease had a larger event-free survival benefit than patients with stages IB to II disease, and patients with tumors expressing PDL 1 at 1% or higher had a larger benefit than patients with PDL 1 expression at less than 1%. With the inclusion of nivolumab in CheckMate-816, the primary benefit in terms of pathological response for patients in stage IIIA was more striking than the benefit for patients in stages IB to II (39). The predictive value of the PDL 1 status may differ in patients with non-metastatic earlier-stage lung cancer with less tumor burden, regardless of the results in metastatic stage IV patients. The Checkmate-816 trial (39) and the NEoverall survivalTAR (32) both demonstrated that increased PDL 1 expression was also connected to more pathologic reactions. Nevertheless, no correlation was discovered between PDL 1 expression and pathogenic response by Shu et al. (27) or the CLMC3 experiment (24).

## Limitations

Given that a few of the researchers chosen for the meta-analysis were not included, a variety bias might have occurred. Nevertheless, the excluded studies did not encounter the necessary standards to be incorporated into the meta-analysis. Moreover, we did not have enough information to determine whether factors such as race and age had an impact on results. Pathological response following neoadjuvant immunotherapy in resectable NSCLCs was the aim of the study. Bias may have increased as a result of the incorporation of incomplete or erroneous data from earlier studies. The individuals’ age, gender, and race were likely sources of bias in addition to their nutritional status. Unintentionally skewed values might arise from incomplete data and unpublished research.

## Conclusions

Individuals with resectable NSCLCs had a significantly higher MPR when comparing neoadjuvant chemo-immunotherapy to neoadjuvant chemotherapy, the objective response rate to non-objective response rate, and PDL 1 ≥ 1% to PDL 1 ≤ 1%. However, no significant difference was found in MPRs between stage III and stage I-II, and squamous cell cancer and non-squamous cell cancer in resectable NSCLCs. To validate this discovery, more studies are required since most of the selected studies had a low sample size (27 out of 35 studies were <100 subjects), and thoughtfulness must be exercised when interrelating with its values. That would have an impact on how significant the evaluated assessments were.

## Data Availability

The original contributions presented in the study are included in the article/Supplementary Material, further inquiries can be directed to the corresponding author.
